# Comparison of Postoperative Analgesic Profiles Between Transversus Abdominis Plane Block and Local Wound Infiltration in Living Donor Kidney Transplantation Recipients: A Propensity Score-Matched Analysis

**DOI:** 10.3390/life15050687

**Published:** 2025-04-23

**Authors:** Min Suk Chae, Kyung Kwan Lee, Jin-Oh Jeong, Wonwoo Jeong, Young Wook Moon, Ji Young Min

**Affiliations:** 1 Department of Anesthesiology and Pain Medicine, Seoul St. Mary’s Hospital, College of Medicine, The Catholic University of Korea, Seoul 06591, Republic of Korea; shscms@catholic.ac.kr; 2 Wake Forest Institute for Regenerative Medicine, Wake Forest School of Medicine, Winston-Salem, NC 27157, USA; kylee@wakehealth.edu (K.K.L.); jijeong@wakehealth.edu (J.-O.J.); wjeong@wakehealth.edu (W.J.); 3 US Research and Production Team, CGBIO USA, Winston-Salem, NC 27101, USA; moon@cgbio.co.kr; 4 Department of Anesthesiology and Pain Medicine, Eunpyeong St. Mary’s Hospital, College of Medicine, The Catholic University of Korea, Seoul 03312, Republic of Korea

**Keywords:** analgesics, opioid, kidney transplantation, living donors, local infiltration anesthesia, postoperative pain, transversus abdominis plane block

## Abstract

Effective postoperative pain management is crucial for optimizing recovery and clinical outcomes in living donor kidney transplantation (LDKT). This retrospective study compared the efficacy and safety of transversus abdominis plane (TAP) block and local wound infiltration (LWI) for postoperative analgesia. A total of 524 LDKT recipients, matched through propensity scoring, were analyzed (262 per group). Pain intensity was assessed using the visual analog scale (VAS) at multiple postoperative time points, while opioid consumption was evaluated based on intravenous patient-controlled analgesia (IV-PCA) usage and rescue fentanyl doses. The TAP block group had significantly lower VAS pain scores at 1, 4, and 8 h postoperatively (*p* < 0.001) and required fewer opioids, as evidenced by reduced IV-PCA usage (55.9 ± 10.2 mL vs. 69.7 ± 18.2 mL; *p* < 0.001) and lower rescue fentanyl doses (67.7 ± 30.6 µg vs. 119.1 ± 71.8 µg; *p* < 0.001). Despite these differences in analgesic efficacy, no significant differences were observed between the groups in terms of postoperative nausea and vomiting or complications such as systemic toxicity and nerve injury. These findings suggest that the TAP block provides more effective early postoperative pain relief and reduces opioid requirements without increasing adverse events. Given its favorable safety profile and effectiveness, the TAP block is a valuable component of multimodal analgesia in LDKT recipients, supporting enhanced recovery while minimizing opioid-related complications.

## 1. Introduction

Living donor kidney transplantation (LDKT) is the preferred treatment for end-stage kidney disease (ESKD), providing better graft survival and overall patient outcomes compared to deceased donor transplantation [1]. Effective postoperative pain management is crucial in this setting, as inadequate analgesia can delay recovery, prolong hospitalization, and increase the risk of complications [2]. While opioids remain a cornerstone of postoperative pain control, their use is linked to significant adverse effects, including dependence, respiratory depression, and potential impairment of graft function [3]. Notably, excessive opioid use in the first year after transplantation has been associated with increased mortality risk and higher rates of all-cause graft failure [4].

LDKT recipients frequently experience moderate to severe postoperative pain due to the surgical incision and intra-abdominal tissue manipulation involved in graft harvesting and implantation [5]. Uncontrolled pain can exacerbate stress responses, negatively impact immune function, and potentially compromise graft survival [6,7,8,9,10]. To mitigate these risks, multimodal analgesia protocols have been developed, emphasizing opioid-sparing strategies [11]. Among regional analgesic techniques, transversus abdominis plane (TAP) block and local wound infiltration (LWI) have gained particular attention [12].

The TAP block is a regional anesthetic technique that involves the injection of a local anesthetic—most commonly ropivacaine, due to its favorable safety profile and lower cardiovascular toxicity [13,14]—into the fascial plane between the internal oblique and transversus abdominis muscles. This technique effectively blocks the sensory nerve supply from T6 to L1, providing analgesia to the anterior abdominal wall and significantly reducing postoperative pain intensity and opioid requirements, particularly during the critical first 24 h after surgery [15]. In contrast, LWI is a simpler and more cost-effective analgesic approach that involves direct injection of local anesthetics, typically ropivacaine, into the tissues surrounding the surgical incision, providing localized postoperative pain relief [16]. In addition to its analgesic effects, LWI may also exert anti-inflammatory benefits through multiple mechanisms, including inhibition of leukocyte activation and migration, suppression of pro-inflammatory cytokines such as interleukin-6 (IL-6) and tumor necrosis factor-alpha (TNF-α), and stabilization of cell membranes [17,18,19]. These actions collectively contribute to reducing local inflammation and tissue injury, thereby enhancing recovery quality.

Although the analgesic effectiveness of both TAP and LWI has been well-established in general abdominal surgeries, their comparative efficacy in the specific context of LDKT remains unclear. Most available studies have focused on non-transplant abdominal or urologic surgical populations [20,21], which may not adequately reflect the unique perioperative challenges of LDKT, such as immunosuppression, altered inflammatory responses, and distinct recovery trajectories [22,23]. For instance, a randomized, double-blinded trial in patients undergoing laparoscopic colorectal cancer resection found no significant differences in postoperative pain scores or recovery outcomes between TAP block and LWI [24]; however, such findings may not be directly applicable to LDKT recipients due to their distinct clinical and immunologic profiles. Addressing this gap is important, as tailored analgesic approaches may improve recovery and reduce opioid-related adverse effects in this vulnerable population. Therefore, a direct comparison of TAP block and LWI in LDKT recipients is warranted to inform evidence-based, transplant-specific postoperative pain management strategies.

Accordingly, we hypothesized that differences in analgesic technique would lead to measurable variations in early postoperative outcomes, including pain scores and opioid use, in this patient population. By comparing TAP block and LWI within a well-defined LDKT cohort, this study aimed to contribute to optimized perioperative care and improved clinical outcomes in kidney transplantation.

## 2. Patients and Methods

### 2.1. Ethical Considerations

This retrospective cohort study was performed at Seoul St. Mary’s Hospital, with ethical approval obtained from the Institutional Review Board and Ethics Committee (approval number: KC22RISI0289) on 6 May 2022. The research strictly adhered to the ethical standards outlined in the Declaration of Helsinki. Due to the retrospective design, the requirement for informed consent was waived by the Ethics Committee. The study’s reporting aligns with the STROBE Statement guidelines to guarantee methodological transparency and accuracy.

### 2.2. Study Population

**Figure 1:**
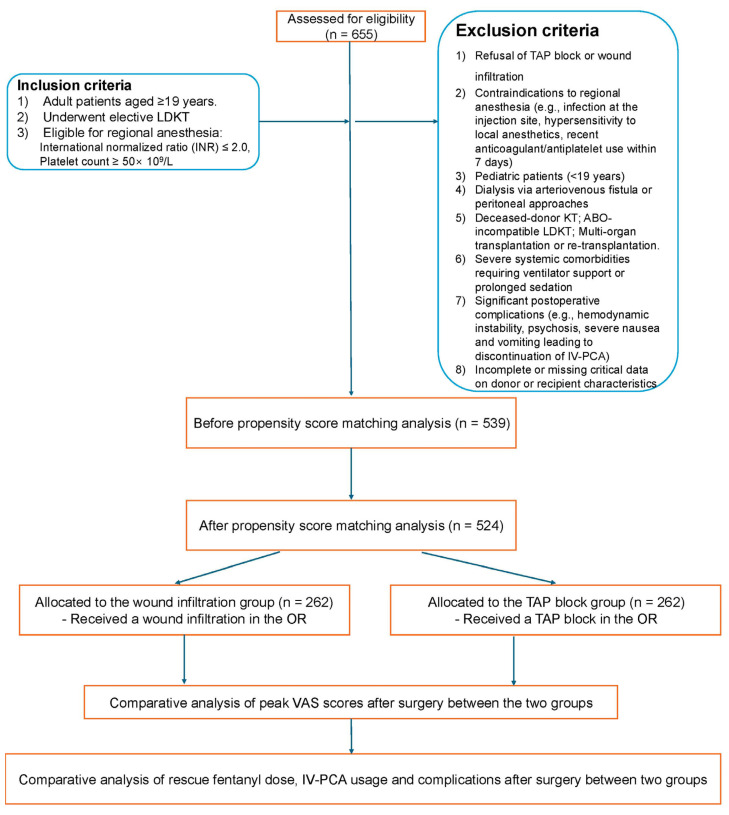
Consort diagram. LDKT, living donor kidney transplantation; INR, international normalized ratio; TAP, transversus abdominis plane; VAS, visual analog scale; OR, operative room; IV-PCA, intravenous patient-controlled analgesia.

This study enrolled adult patients aged 19 years or older with an international normalized ratio (INR) of ≤2.0 and a platelet count of ≥50 × 10^9^/L [25], who underwent elective LDKT at our institution between March 2017 and March 2022. Patients were excluded if they declined TAP block or wound infiltration, had contraindications to regional anesthesia (e.g., active injection site infections or hypersensitivity to local anesthetics), had used anticoagulant or antiplatelet medications within seven days prior to surgery, or were pediatric patients (<19 years) or dialysis recipients using arteriovenous fistula or peritoneal approaches due to altered pharmacokinetics of local anesthetics [26]. Additional exclusions included deceased-donor KT, ABO-incompatible LDKT, multi-organ transplants, re-transplantation, and early postoperative re-operations. Patients with severe systemic comorbidities necessitating ventilator support or prolonged sedation, significant postoperative complications such as hemodynamic instability, psychosis, or severe nausea/vomiting leading to discontinuation of intravenous patient-controlled analgesia (IV-PCA), or incomplete or missing critical data were also excluded. From the initial cohort of 655 patients, 539 met the inclusion criteria after applying the exclusion parameters. Propensity score (PS) matching was performed to balance baseline characteristics, resulting in 524 patients evenly divided between the wound infiltration group (n = 262) and the TAP block group (n = 262) for final analysis (Figure 1).

### 2.3. Surgery and General Anesthesia

The surgical procedure for LDKT began with a pararectal inverted J-shaped curvilinear incision, also known as a hockey-stick incision, to provide access to the right pelvic fossa. During surgery, standard hand-held retractors were routinely utilized to maintain optimal surgical exposure throughout the procedure; self-retaining retractors were not used in any case. The graft was prepared on a back table, followed by end-to-side vascular anastomoses between the graft’s renal artery and vein and the recipient’s external iliac artery and vein. These anastomoses were performed using 6-0 Prolene non-absorbable monofilament sutures (Ethicon, Somerville, NJ, USA). Subsequently, ureteroneocystostomy was performed using the Lich-Grègoir technique, with the placement of a double-J stent (INLAY ureteral stent; Bard Medical, Covington, GA, USA) to ensure proper urinary drainage. After achieving meticulous hemostasis and thoroughly reassessing the vascular anastomoses and renal pedicle, closed drains were placed, and the surgical incision was securely closed [27,28].

Upon entering the operating room, patients were connected to standard monitoring devices. Anesthesia induction was achieved with intravenous (IV) propofol (Fresofol MCT 2%, Fresenius Kabi, Graz, Austria) at 1.5–2 mg/kg and rocuronium (Esmeron, MSD, Kenilworth, NJ, USA) at 0.8–1 mg/kg. Maintenance of balanced general anesthesia was accomplished using inhaled desflurane (Suprane, Baxter Healthcare, Deerfield, IL, USA) at concentrations of 3–6%, combined with IV remifentanil (Ultiva, GlaxoSmithKline, Brentford, UK) at 0.01–0.2 µg/kg/min. A Bispectral index (BIS) of 40–60 was targeted to ensure appropriate anesthetic depth. To optimize urinary flow, mannitol (Daihan Pharm. Co., Seoul, Republic of Korea) at 20–50 g was administered intravenously 10 min before graft reperfusion, followed by furosemide (Handok Inc., Seoul, Republic of Korea) at 20–40 mg after reperfusion. Intraoperative fluid management involved the infusion of crystalloids, including 0.9% normal saline (CJ Healthcare, Seoul, Republic of Korea) and Plasma Solution-A (HK inno.N Corp., Seoul, Republic of Korea), tailored to patient weight and surgical demands. Hydration volumes of 50–100 mL/kg were used to maintain central venous pressure at 10–15 mmHg, ensuring sufficient renal perfusion and offsetting urinary losses following vascular declamping. Neuromuscular blockade was reversed at the end of the procedure using sugammadex (Bridion, MSD, Kenilworth, NJ, USA) at 4 mg/kg. Extubation was performed after verifying airway patency and achieving a train-of-four ratio of ≥90%, confirming adequate recovery of neuromuscular function [29].

### 2.4. Local Wound Infiltration and TAP Block in the Operating Room

The sequence of LWI and TAP block followed the routine surgical workflow established for the procedure. Wound infiltration was carried out immediately prior to wound closure by a single experienced surgeon who was not involved in the study, ensuring consistent and unbiased application. A total of 20 mL of 0.375% ropivacaine (Mitsubishi Tanabe Pharma, Osaka, Japan) was administered incrementally along the surgical incision using aseptic technique. The infiltration targeted the subcutaneous and musculofascial planes to provide comprehensive analgesia. A sterile 22-gauge needle was systematically advanced along the incision under direct visualization, delivering small, incremental doses at regular intervals. Aspiration was performed before each injection to avoid intravascular administration, and hydro-dissection was employed when necessary to confirm proper placement in the targeted tissue plane and enhance anesthetic spread. This careful and systematic approach ensured even distribution of the anesthetic across the incision site, reducing the risk of complications and maximizing postoperative pain relief. The procedure was completed before wound closure to optimize analgesic effectiveness [16].

A TAP block was performed on the surgical side immediately following the procedure by a single expert anesthesiologist who was not involved in the study, ensuring objectivity. The block was administered using a linear ultrasound probe (Affiniti 70 Ultrasound System, Philips, Amsterdam, The Netherlands) positioned along the mid-axillary line between the iliac crest and the lower costal margin. Under ultrasound guidance, the three layers of the abdominal wall—external oblique, internal oblique, and transversus abdominis—were clearly visualized. A sterile 21-gauge, 8.5 mm block needle (Echoplex; Vygon Co., Paris, France) was advanced using an in-plane technique with precision, ensuring continuous visualization of the needle tip throughout the procedure. The needle was inserted medially to laterally, targeting the fascial plane between the internal oblique and transversus abdominis muscles. Correct placement was confirmed via hydro-dissection using a small test dose of normal saline. Subsequently, 20 mL of 0.375% ropivacaine (Mitsubishi Tanabe Pharm. Co., Osaka, Japan) was incrementally injected under real-time ultrasound guidance to ensure even distribution within the fascial plane and to minimize risks such as intravascular injection. This meticulous technique ensured accurate placement of the anesthetic, optimizing postoperative pain relief while maintaining a high level of safety [30].

### 2.5. Adjuvant Strategies for Postoperative Pain Management

The preemptive analgesic protocol for all patients included the slow intravenous infusion of 1 g paracetamol (Woosung Pharm. Co., Suwon, Gyeonggi-do, Republic of Korea) and 20 mg nefopam (Myungmoon Pharm. Co., Seoul, Republic of Korea) during wound closure to provide non-opioid pain relief. To prevent postoperative nausea and vomiting (PONV), a preemptive intravenous injection of 0.075 mg palonosetron hydrochloride (Aloxi; CJ Healthcare, Seoul, Republic of Korea) was administered. For patients experiencing breakthrough PONV, ondansetron 4 mg (Zofran; Hana Pharm, Seoul, Republic of Korea) was administered intravenously postoperatively as needed.

Following LDKT, all patients were provided with intravenous patient-controlled analgesia (IV-PCA) using the AutoMed 3200 device (Acemedical, Seoul, Republic of Korea). The IV-PCA solution consisted of 1000 µg fentanyl (Dai Han Pharma. Co., Seoul, Republic of Korea) and 0.3 mg ramosetron (Boryung Pharma. Co., Seoul, Republic of Korea), diluted in normal saline to a total volume of 100 mL. The device was configured to deliver a continuous basal infusion of 1 mL per hour, with an optional 1 mL bolus available at a 10 min lockout interval. Patients were instructed to administer a bolus dose when their pain intensity, measured on the visual analog scale (VAS), reached a score of 4 or higher, where 0 represents no pain and 10 signifies the worst possible pain. This setup allowed for both consistent pain management and patient-controlled relief for breakthrough pain.

Postoperative pain intensity was evaluated using the visual analog scale (VAS), a 10-cm horizontal line anchored by descriptors where 0 indicates no pain and 10 indicates the worst pain imaginable. Pain assessments were conducted by attending nurses who were not involved in the study, immediately after surgery in the post-anesthesia care unit (PACU), and subsequently at 1, 4, 8, 12, and 24 h postoperatively in the intensive care unit (ICU). Patients marked a point on the VAS line corresponding to their perceived pain intensity at each time point. Patients who reported inadequate pain relief despite IV-PCA settings received an additional 50 µg of intravenous fentanyl as a rescue analgesic, administered by an attending physician who was also not associated with the study. Cumulative IV-PCA usage and rescue fentanyl doses were carefully monitored and recorded at each assessment point to ensure accurate tracking of opioid consumption. Patients were closely observed for potential opioid-related complications, such as PONV and respiratory depression, enabling prompt intervention when necessary.

### 2.6. Outcome Measures

This study primarily evaluated postoperative pain intensity, measured using the VAS, and total opioid consumption, including rescue fentanyl doses and IV-PCA usage, within the first 24 h after surgery. Pain levels were assessed at specific intervals: immediately after surgery, and at 1, 4, 8, 12, and 24 h postoperatively. Secondary outcomes included the incidence of opioid-related complications, such as PONV. These measures aimed to compare the efficacy of wound infiltration and TAP block in managing postoperative pain and minimizing opioid-related side effects. Outcome data were collected by trained nurses uninvolved in the study to ensure objectivity and accuracy. The timing and quantity of opioid administration, along with the presence of opioid-related adverse effects including PONV, respiratory depression, sedation, pruritus, and urinary retention, were systematically recorded to facilitate comprehensive analysis.

### 2.7. Clinical Variables

The clinical variables analyzed in this study included preoperative, intraoperative, and donor/graft-related factors to ensure baseline equivalence between the wound infiltration and TAP block groups. Preoperative variables comprised patient demographics, such as sex, age, and body mass index (BMI), as well as comorbidities like diabetes mellitus and hypertension. Dialysis duration prior to surgery was also recorded. Cardiac function was evaluated using echocardiographic parameters, including left ventricular ejection fraction (EF), left ventricular mass index (LVMI), and E/e′ ratio. Cardiac electrical activity was assessed via the corrected QT interval measured by electrocardiography. Laboratory assessments included white blood cell (WBC) count, neutrophil and lymphocyte percentages, hemoglobin, glucose, albumin, aspartate aminotransferase (AST), alanine aminotransferase (ALT), electrolytes (sodium, potassium, chloride), platelet count, INR, creatinine, B-type natriuretic peptide (BNP), and troponin I/T levels. Intraoperative variables included operative time, hourly fluid infusion rates, and vital signs such as systolic blood pressure (SBP), diastolic blood pressure (DBP), and heart rate. Donor and graft-related factors analyzed included donor sex, age, BMI, graft laterality (left or right kidney), graft weight, ischemic time, and hemoglobin levels. All variables were carefully documented and validated to reduce the risk of confounding and ensure accurate comparisons. PS matching was utilized to balance baseline clinical, demographic, and perioperative characteristics between the TAP block and LWI groups. The PS was calculated based on logistic regression, including variables such as age, sex, BMI, comorbidities (diabetes mellitus and hypertension), dialysis duration, echocardiographic parameters, laboratory findings, intraoperative fluid management, and donor-related factors. Matching was performed using a 1:1 nearest-neighbor algorithm without replacement and a caliper width of 0.2 standard deviations of the logit of the propensity score. Rigorous data collection involved standardized electronic medical record extraction, and verification protocols included independent double-checking by two experienced researchers to ensure data integrity and reliability.

### 2.8. Statistical Analysis

As this was a retrospective observational cohort study, no formal a priori power analysis was conducted. Instead, we utilized all available data from eligible patients who underwent LDKT at our institution between March 2017 and March 2022. After applying inclusion and exclusion criteria, PS matching was performed to balance baseline characteristics and minimize selection bias, resulting in a final matched cohort of 524 patients (262 patients per group). To confirm the adequacy of our sample size, a post hoc power analysis was conducted based on the observed differences in primary outcomes between the TAP block and LWI groups. Specifically, using the mean VAS pain scores at 4 h postoperatively (TAP: 3.6 ± 1.0 vs. LWI: 4.7 ± 1.4), the calculated observed effect size (Cohen’s d) was 0.91. Using this effect size, an alpha level of 0.05 (two-tailed), and the current sample size of 262 per group, this study achieved statistical power exceeding 99%. This calculation confirms that the sample size was sufficient to detect clinically significant differences in postoperative pain between the two analgesic techniques. The power calculation was conducted using G*Power software (version 3.1, Heinrich Heine Universität, Düsseldorf, Germany) [31].

The normality of continuous variables was assessed using the Shapiro–Wilk test. Variables following a normal distribution were compared using Student’s *t*-test, while those not meeting normality assumptions were analyzed using the Mann–Whitney U test. Categorical variables were compared using the chi-squared test or Fisher’s exact test, as appropriate. Postoperative pain intensity (VAS scores) across time points was evaluated using repeated-measures ANOVA, following verification of sphericity (Mauchly’s test) and homogeneity of variances (Levene’s test). Pairwise comparisons were conducted using paired *t*-tests with Bonferroni correction to adjust for multiple testing. Propensity score matching was conducted using a 1:1 nearest-neighbor algorithm without replacement and a caliper of 0.2 standard deviations of the logit of the propensity score. Matching quality was assessed using standardized mean differences (SMD), with SMD < 0.2 considered acceptable. Categorical variables were expressed as frequencies and percentages, and continuous variables as mean with standard deviations (SD) or medians with interquartile ranges (IQR), based on their distribution. All statistical analyses were performed using R software (version 2.10.1; R Foundation for Statistical Computing, Vienna, Austria), and *p*-values < 0.05 were considered statistically significant. Graphical outputs were generated using Microsoft Excel (Microsoft Corporation, Redmond, WA, USA).

## 3. Results

### 3.1. Baseline Characteristics of the Study Population

Table 1 provides an overview of the baseline demographic, clinical, and perioperative characteristics of patients in the wound infiltration and TAP block groups, both before and after PS matching. Prior to matching, 270 patients were assigned to the wound infiltration group and 269 to the TAP block group. A significant difference was observed in the prevalence of diabetes mellitus, which was higher in the TAP block group compared to the wound infiltration group (41.6% vs. 31.1%, *p* = 0.011). Other baseline variables, including age, sex, body mass index (BMI), dialysis duration, echocardiographic parameters (ejection fraction, LVMI, E/e′ ratio), and laboratory findings, showed no significant differences between the groups.

After PS matching, 262 patients were included in each group, resulting in balanced baseline characteristics. No significant differences were identified between the groups in terms of age, sex, BMI, comorbidities (diabetes mellitus and hypertension), or dialysis duration. Cardiac function parameters, including ejection fraction, LVMI, and E/e′ ratio, as well as laboratory markers such as WBC count, hemoglobin, glucose, albumin, AST, ALT, electrolytes, and creatinine, were also comparable. Furthermore, the corrected QT interval, an indicator of cardiac electrical activity, demonstrated no significant variation between the groups after matching, confirming the effectiveness of PS matching in achieving baseline comparability.

### 3.2. Postoperative Pain and Opioid Requirements

**Figure 2:**
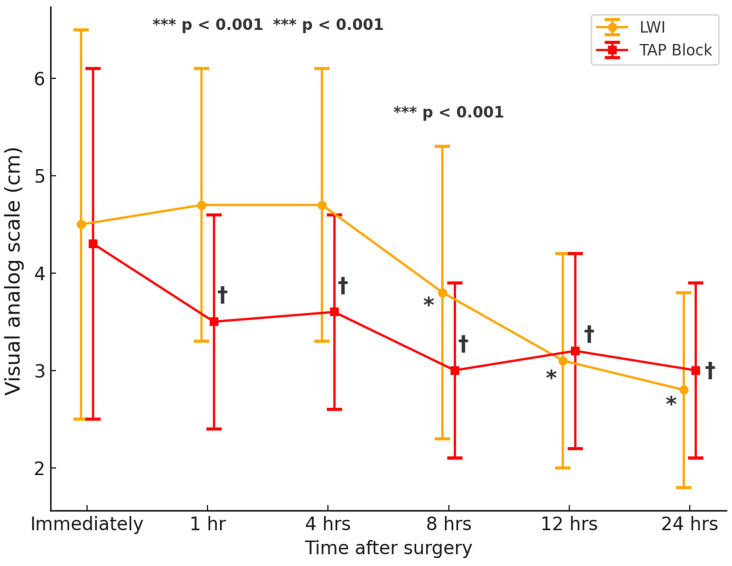
Comparison of visual analog scale between the TAP (transversus abdominis plane) block group and LWI (local wound infiltration) groups over time. The vertical axis represents time, while the horizontal axis represents the visual analog scale. Data are presented as mean ± standard deviation. The TAP (transversus abdominis plane) block group is shown in red, and the LWI (local wound infiltration) group is shown in orange. ^†^ *p* < 0.001 compared to the values immediately after surgery in the TAP block group. * *p* < 0.001 compared to the values immediately after surgery in the LWI group. *** *p* < 0.001 compared to the values between TAP and LWI groups.

Table 2 and Figure 2 highlight the postoperative pain scores, opioid consumption, and complications in the wound infiltration and TAP block groups. The VAS pain scores showed significant differences between the groups at various postoperative intervals. While the mean VAS score immediately after surgery was similar (wound infiltration: 4.5 ± 2.0 vs. TAP block: 4.3 ± 1.8; *p* = 0.202), the TAP block group demonstrated significantly lower pain scores at 1 h (3.5 ± 1.1 vs. 4.7 ± 1.4; *p* < 0.001), 4 h (3.6 ± 1.0 vs. 4.7 ± 1.4; *p* < 0.001), and 8 h (3.0 ± 0.9 vs. 3.8 ± 1.5; *p* < 0.001) postoperatively. By 12 and 24 h after surgery, VAS scores were comparable, with no statistically significant differences observed.

Opioid consumption on postoperative day 1 (POD 1) was significantly lower in the TAP block group. The total rescue fentanyl dose was reduced in the TAP block group compared to the wound infiltration group (67.7 ± 30.6 µg vs. 119.1 ± 71.8 µg; *p* < 0.001). Similarly, intravenous patient-controlled analgesia (IV-PCA) usage was significantly lower in the TAP block group (55.9 ± 10.2 mL vs. 69.7 ± 18.2 mL; *p* < 0.001).

The incidence of postoperative nausea and vomiting (PONV) related to opioid use was lower in the TAP block group (13.4%) than in the wound infiltration group (19.1%), although this difference did not reach statistical significance (*p* = 0.075).

### 3.3. Complications

Complications associated with local anesthetics, including systemic toxicity, hematoma formation, and injection site infection, were systematically monitored through thorough postoperative assessments, clinical records review, laboratory tests, and patient follow-up data during the study period. Importantly, none of these complications were identified in either group, underscoring the safety of both techniques when performed under controlled conditions.

For opioid-related adverse events, no cases of respiratory depression or sedation requiring medical intervention were observed. Minor side effects, including pruritus and urinary retention, showed no significant differences between the groups and were deemed clinically insignificant.

## 4. Discussion

This study demonstrates that the TAP block provided more effective early postoperative analgesia than LWI, as evidenced by lower VAS scores at 1, 4, and 8 h postoperatively and reduced opioid consumption. Patients who received a TAP block required significantly less opioid analgesia, including decreased IV-PCA usage and fewer rescue fentanyl doses. These findings suggest that the TAP block contributes to improved acute postoperative pain management in the early recovery period.

The superior early postoperative analgesic effect observed in this study aligns with previous reports in abdominal and urologic surgeries [20,21]. While such studies confirm the value of TAP block in general surgical settings, the present analysis provides direct comparative data from LDKT recipients—a population with distinct perioperative needs and heightened sensitivity to opioid-related adverse effects. By evaluating early postoperative pain scores and opioid consumption within a propensity score-matched cohort, this study enhances the evidence base for analgesic decision-making tailored to LDKT. These findings expand upon prior data by offering LDKT-specific evidence supporting the role of TAP block as an effective analgesic modality within transplant-focused enhanced recovery protocols, and they further justify its broader integration into opioid-sparing perioperative strategies for this population.

This analgesic advantage of the TAP block can be attributed to both its anatomical and pharmacokinetic properties. Ropivacaine was selected for the TAP block based on its favorable safety profile and pharmacodynamic characteristics [32,33,34]. It provides prolonged analgesia while exhibiting lower cardiotoxicity and central nervous system toxicity compared to other long-acting local anesthetics, such as bupivacaine. Moreover, its preferential sensory blockade with minimal motor impairment facilitates early postoperative mobilization and recovery, which is particularly beneficial in kidney transplant recipients. By delivering local anesthetic within the transversus abdominis plane, the TAP block results in slow systemic absorption and sustained blockade of the T6–L1 sensory nerves, providing prolonged and broader analgesia to the anterior and lateral abdominal walls. In contrast, LWI primarily targets nociceptors at the incision site and is associated with faster vascular absorption, which may limit both the duration and extent of analgesia [16,30,35]. These differences likely explain the lower early postoperative pain scores and reduced opioid consumption observed in the TAP block group. Importantly, the opioid-sparing effect has additional clinical implications beyond pain relief, particularly in reducing opioid-related adverse events such as PONV [36].

The opioid-sparing effect of the TAP block is achieved through two mechanisms: (1) prolonged sensory blockade minimizes breakthrough pain and reduces opioid-based rescue analgesia [37], and (2) attenuation of central sensitization may help prevent the development of prolonged pain states, thereby decreasing overall opioid requirements [38]. While opioid reduction can help mitigate PONV, a clinically significant reduction typically requires a 30–50% decrease in opioid consumption [39]. In this study, although opioid use was significantly lower in the TAP block group, the impact on PONV did not reach statistical significance. This suggests that additional factors, such as patient-specific characteristics (e.g., female sex, non-smoking status, history of PONV) and anesthetic-related variables, may have influenced PONV outcomes [40,41]. A multimodal approach integrating regional anesthesia, pharmacologic prophylaxis, and individualized perioperative management remains essential for optimizing patient recovery [42].

Despite its advantages, the TAP block has limitations in both duration and coverage. Some kidney transplant recipients may experience pain that extends beyond the typical dermatomal coverage area, resulting in suboptimal analgesia. In such cases, bilateral TAP blocks or alternative regional anesthesia techniques—such as quadratus lumborum (QL) blocks or continuous catheter-based TAP blocks—may provide more comprehensive pain control and enhance patient comfort during the early postoperative period [42,43,44]. Among these, catheter-based TAP blocks have demonstrated superior efficacy in prolonging analgesic duration and improving patient satisfaction [45,46]. Additionally, because the analgesic effect of the TAP block typically wanes after approximately 12 h, a multimodal strategy incorporating NSAIDs, acetaminophen, and early administration of oral analgesics is recommended to manage rebound pain and maintain adequate analgesia [47]. Furthermore, as patient-specific factors such as age, BMI, and sex can influence block efficacy, individualized analgesic strategies should be considered. In particular, for obese patients, ultrasound guidance is recommended to ensure accurate anesthetic delivery and optimize pain relief [48,49,50,51].

Overall, these findings highlight the potential of the TAP block as an effective pain management strategy for LDKT recipients, particularly in the early postoperative period. Both the TAP block and LWI demonstrated a low incidence of complications, such as systemic toxicity, nerve injury, and injection site infections, reinforcing their favorable safety profile. Compared to LWI, the TAP block provides more prolonged and widespread analgesia, supporting its incorporation into enhanced recovery after surgery (ERAS) protocols [24]. These protocols prioritize opioid-sparing strategies and integrate regional anesthesia techniques, including TAP block and LWI, alongside scheduled administration of non-opioid analgesics such as acetaminophen and nefopam, particularly in settings where NSAID use is restricted [52,53,54].

Additionally, the type and method of surgical retraction may influence postoperative pain and tissue trauma. In this study, only hand-held retractors were used, which require intermittent repositioning and manual pressure. This approach may reduce the risk of continuous tissue ischemia compared to self-retaining retractors that apply sustained localized pressure [55]. Previous studies have shown that prolonged use of self-retaining retractors can increase the risk of nerve compression injuries, such as femoral neuropathy, in kidney transplantation, potentially resulting in severe postoperative pain and delayed recovery [56,57,58]. In contrast, intermittent retraction with hand-held retractors allows periodic relief of tissue tension and restoration of perfusion, potentially mitigating such risks [55,57]. Therefore, the exclusive use of hand-held retractors in our cohort may have contributed to minimizing retractor-related postoperative pain and associated complications. Comparative studies are needed to evaluate postoperative outcomes associated with different retractor types in kidney transplantation.

While these findings support the use of the TAP block as a valuable analgesic technique, certain limitations should be considered. First, as a retrospective cohort study, potential selection bias and confounding factors remain, despite the use of propensity score matching [59]. Second, pain assessment was limited to resting pain, without evaluating dynamic pain during movement or coughing, which is essential for assessing functional recovery and mobilization. Pain intensity measurements primarily reflected peak levels prior to analgesic administration, typically at rest. Due to the retrospective design of this study, separate assessments of dynamic pain, such as pain during coughing or mobilization, could not be conducted. Dynamic pain influences early ambulation and pulmonary function, and inadequate control may lead to delayed recovery and increased postoperative complications [60]. Third, this study was conducted at a single center with a relatively homogeneous patient population, limiting the external validity of the findings. Institutional practices, regional demographics, and clinical protocols may differ across settings, potentially affecting the generalizability of the results [61]. Finally, rebound pain, which may occur as the analgesic effects of the TAP block wear off, was not specifically evaluated, leaving an important gap in understanding the transition of pain relief [47,62]. Future studies should explore strategies to mitigate rebound pain, such as adjunctive use of continuous regional anesthesia techniques or preemptive multimodal analgesic regimens. Prospective, multicenter studies incorporating long-term pain assessments, dynamic pain evaluations, and alternative regional analgesic techniques are needed to refine and optimize postoperative pain management strategies for LDKT recipients.

## 5. Conclusions

This study highlights the clinical utility of the TAP block for postoperative pain management in LDKT recipients. Compared to LWI, TAP block was associated with lower early pain scores and reduced opioid consumption, thereby supporting its use as part of multimodal analgesic strategies. Its broad and prolonged analgesic coverage offers practical advantages for early recovery, particularly in the setting of enhanced recovery protocols. However, the limited duration of a single-shot TAP block underscores the need for adjunctive measures to sustain analgesia. Given their established safety profiles, both the TAP block and LWI remain viable options, with technique selection best guided by institutional resources and patient-specific factors. Future research should focus on optimizing the duration of effect, incorporating patient-reported outcomes, and developing personalized, transplant-specific pain management pathways.

## Figures and Tables

**Table 1:** Demographic variables in the local wound infiltration (LWI) and transversus abdominis plane (TAP) block groups before and after propensity score matching.

	Before PS Matching				After PS Matching			
Group	TAP Block (*n* = 269)	LWI (*n* = 270)	*p* Value	SD	TAP Block (*n* = 262)	LWI (*n* = 262)	*p* Value	SD
***Preoperative variables***								
Sex; n (%)	138 (51.3%)	124 (45.9%)	0.212	0.107	128 (48.9%)	144 (55.0%)	0.162	0.122
Age; years	52.0 (41.5–59.0)	50.0 (40.0–57.0)	0.124	0.121	51.5 (41.8–59.0)	50.0 (40.8–57.0)	0.141	0.117
BMI; kg/m^2^	22.8 (20.6–26.0)	22.9 (20.4–25.4)	0.676	0.071	22.9 (20.6–26.1)	23.0 (20.4–25.5)	0.643	0.073
Diabetes mellitus; n (%)	112 (41.6%)	84 (31.1%)	0.011	0.213	105 (40.1%)	84 (32.1%)	0.056	0.193
Hypertension; n (%)	146 (54.3%)	132 (48.9%)	0.211	0.108	142 (54.2%)	128 (48.9%)	0.221	0.107
Dialysis period; day	1.0 (0.0–7.5)	1.0 (0.0–11.0)	0.900	−0.081	1.0 (0.0–6.0)	0.5 (0.0–10.0)	0.937	−0.082
Echocardiography								
Ejection fraction; %	62.0 (58.7–64.8)	62.0 (57.3–64.6)	0.314	0.086	62.0 (58.8–64.9)	62.0 (57.9–64.7)	0.481	0.038
LVMI; g/m^2^	119.1 (101.0–140.3)	119.1 (102.0–144.0)	0.376	−0.109	119.0 (99.1–140.2)	119.1 (101.1–142.6)	0.435	−0.103
E/e’ ratio	10.3 (8.7–12.9)	10.0 (7.8–12.7)	0.170	0.005	10.3 (8.7–12.6)	10.0 (7.8–12.5)	0.156	0.004
Corrected QT interval; ms	450.0 (431.0–469.5)	452.0 (432.0–475.0)	0.431	−0.069	450.0 (430.8–469.3)	452.0 (432.0–473.3)	0.456	−0.067
Hourly urine output; mL/kg/h	18.8 (12.5–25.0)	17.1 (10.4–25.0)	0.150	0.131	18.8 (12.5–25.0)	17.9 (10.4–25.0)	0.187	0.119
Laboratory variables								
WBC; ×10^9^/L	6.2 (4.8–8.1)	6.3 (4.8–7.8)	0.983	−0.026	6.2 (4.8–8.1)	6.3 (4.8–7.8)	0.942	−0.025
Neutrophil; %	70.5 (61.6–85.4)	67.2 (60.7–82.9)	0.284	0.099	70.3 (61.5–85.2)	67.4 (60.7–82.9)	0.419	0.079
Lymophocyte; %	18.4 (10.9–25.2)	19.7 (11.6–26.3)	0.211	−0.106	18.7 (10.9–25.1)	19.7 (11.6–26.2)	0.313	−0.087
Hemoglobin; g/dL	10.7 (9.6–11.5)	10.6 (9.4–11.6)	0.508	0.025	10.7 (9.6–11.5)	10.6 (9.5–11.6)	0.653	0.016
Glucose; mg/dL	121.0 (95.5–155.0)	118.0 (95.0–147.0)	0.599	0.042	120.0 (95.0–151.5)	119.0 (95.0–147.3)	0.762	0.024
Albumin; g/dL	4.1 (3.8–4.3)	4.1 (3.8–4.3)	0.540	0.053	4.1 (3.8–4.3)	4.1 (3.8–4.3)	0.772	0.026
AST; U/L	17.0 (14.0–23.0)	17.0 (14.0–22.0)	0.826	0.018	17.0 (14.0–23.0)	17.0 (14.0–22.0)	0.914	0.019
ALT; U/L	14.0 (10.0–20.0)	13.0 (10.0–19.0)	0.235	0.057	14.0 (10.0–20.0)	13.0 (10.0–18.3)	0.161	0.068
Sodium; mmol/L	138.0 (135.0–140.0)	138.0 (135.0–140.0)	0.891	0.021	138.0 (135.0–140.0)	138.0 (135.0–140.0)	0.906	0.015
Potassium; mmol/L	4.7 (4.2–5.2)	4.7 (4.3–5.2)	0.577	−0.025	4.7 (4.2–5.2)	4.7 (4.3–5.2)	0.382	−0.055
Chloride; mmol/L	100.0 (97.0–104.0)	100.0 (96.0–104.3)	0.967	0.011	100.0 (97.0–104.3)	100.0 (96.0–105.0)	0.978	0.006
Platelet count; ×10^9^/L	178.0 (140.5–218.0)	178.5 (141.0–230.3)	0.466	−0.114	178.0 (140.8–218.0)	180.0 (141.0–230.3)	0.394	−0.122
INR	1.00 (0.96–1.05)	1.01 (0.97–1.07)	0.117	−0.173	1.00 (0.96–1.05)	1.01 (0.96–1.07)	0.200	−0.172
Creatinine; mg/dL	7.1 (6.0–9.1)	7.6 (5.9–9.3)	0.371	−0.06	7.1 (6.0–9.1)	7.5 (5.9–9.2)	0.508	−0.037
BNP; pg/mL	78.7 (31.6–177.2)	82.4 (36.1–218.7)	0.166	−0.391	75.9 (31.2–176.1)	77.3 (34.8–197.9)	0.302	−0.193
Troponin I; pg/mL	21.4 (11.1–44.6)	20.5 (10.4–46.3)	0.631	−0.15	21.1 (11.0–44.5)	20.4 (10.4–46.3)	0.588	−0.056
Troponin T; ng/mL	0.04 (0.02–0.06)	0.03 (0.02–0.05)	0.237	0.014	0.04 (0.02–0.06)	0.03 (0.02–0.05)	0.247	0.006
***Intraoperative variables***								
Operation time; min	225.0 (195.0–255.0)	225.0 (190.0–260.0)	0.677	0.015	225.0 (195.0–255.0)	221.0 (190.0–260.0)	0.482	0.043
Hourly fluid infusion; mL/kg/h	9.0 (7.4–11.4)	9.3 (7.3–11.6)	0.827	−0.062	9.0 (7.2–11.4)	9.3 (7.3–11.7)	0.417	−0.091
Average of vital signs								
SBP; mmHg	132.0 (124.0–141.0)	130.0 (120.0–140.0)	0.033	0.178	131.0 (123.8–140.0)	130.0 (120.0–140.0)	0.069	0.147
DBP; mmHg	80.0 (80.0–90.0)	80.0 (79.0–90.0)	0.256	0.092	80.0 (80.0–90.0)	80.0 (80.0–90.0)	0.411	0.061
Heart rate beats/min	80.0 (73.0–88.0)	80.0 (73.0–88.0)	0.583	0.042	80.0 (73.0–88.0)	80.0 (73.0–88.3)	0.491	0.039
***Donor/graft variables***								
Sex; n (%)	163 (60.6%)	176 (65.2%)	0.270	−0.094	161 (61.5%)	169 (64.5%)	0.469	−0.062
Age; years	51.0 (38.0–59.0)	51.0 (41.0–57.0)	0.941	−0.023	51.0 (38.0–59.0)	51.0 (41.0–57.0)	0.991	−0.026
BMI; kg/m^2^	23.5 (21.9–26.1)	23.6 (21.7–25.6)	0.602	0.068	23.5 (21.9–26.1)	23.7 (21.8–25.7)	0.811	0.049
Left kidney graft; n (%)	174 (64.7%)	162 (60.0%)	0.262	−0.098	93 (35.5%)	105 (40.1%)	0.280	−0.096
Graft weight; g	174.0 (150.0–208.0)	178.0 (150.0–204.5)	0.810	−0.001	174.0 (150.0–208.0)	178.0 (152.0–206.5)	0.601	−0.022
Graft ischemic time; min	54.0 (45.0–67.5)	55.0 (43.8–67.3)	0.885	−0.041	54.5 (45.0–68.0)	55.0 (43.0–67.0)	0.803	0.005
Hemoglobin; g/dL	13.9 (12.9–15.1)	13.7 (12.8–14.9)	0.540	0.035	13.8 (12.9–15.1)	13.7 (12.8–14.9)	0.626	0.027

Values are expressed as median (interquartile) and number (percentage). Abbreviations: SD, standard deviation; TAP, transversus abdominis plane; LWI, local wound infiltration; BMI, body mass index; LVMI, left ventricular mass index; WBC, white blood cell count; AST, aspartate aminotransferase; ALT, alanine aminotransferase; INR, international normalized ratio; BNP, B-type natriuretic peptide; SBP, systolic blood pressure; DBP, diastolic blood pressure.

**Table 2:** Postoperative pain degree, opioid requirement, and complications in propensity score-matched patients.

Group	TAP Block (*n* = 262)	LWI (*n* = 262)	*p* Value
**Visual analog scale (cm)**			
Immediately after surgery	4.3 ± 1.8	4.5 ± 2.0	0.202
1 h after surgery	3.5 ± 1.1 ^†^	4.7 ± 1.4	<0.001 ***
4 h after surgery	3.6 ± 1.0 ^†^	4.7 ± 1.4	<0.001 ***
8 h after surgery	3.0 ± 0.9 ^†^	3.8 ± 1.5 *	<0.001 ***
12 h after surgery	3.2 ± 1.0 ^†^	3.1 ± 1.1 *	0.481
24 h after surgery	3.0 ± 0.9 ^†^	2.8 ± 1.0 *	0.052
**Fentanyl infusion for POD 1**			
Rescue dose (μg)	67.7 ± 30.6	119.1 ± 71.8	<0.001 ***
IV-PCA (mL)	55.9 ± 10.2	69.7 ± 18.2	<0.001 ***
**Opioid-related PONV**	35 (13.4%)	50 (19.1%)	0.075

Abbreviations: TAP, transversus abdominis plane; LWI, local wound infiltration; POD, postoperative day; IV-PCA, intravenous patient-controlled analgesia; PONV, postoperative nausea and vomiting. Values are expressed as mean (±standard deviation) and number (percentage). ^†^ *p* < 0.001 compared to the values immediately after surgery in the TAP block group. * *p* < 0.001 compared to the values immediately after surgery in the LWI group. *** *p* < 0.001 compared to the values between TAP and LWI group.

## Data Availability

Data are contained within the article. The datasets used and analyzed in the current study are available from the corresponding author upon reasonable request.
